# Subwavelength Interferometric Control of Absorption in Three-port Acoustic Network

**DOI:** 10.1038/s41598-018-30287-y

**Published:** 2018-08-17

**Authors:** O. Richoux, V. Achilleos, G. Theocharis, I. Brouzos

**Affiliations:** 1 Le Mans University, LAUM UMR CNRS 6613, Av. O. Messiaen, 72085 Le Mans, France; 2 Department of Physics, University of Athens, 15771 Athens, Greece

## Abstract

Utilizing the effect of losses, we show that symmetric 3-port devices exhibit coherent perfect absorption of waves and we provide the corresponding conditions on the reflection and transmission coefficients. Infinite combinations of asymmetric inputs with different amplitudes and phase at each port as well as a completely symmetric input, are found to be perfectly absorbed. To illustrate the above we study an acoustic 3-port network operating in a subwavelength frequency both theoretically and experimentally. In addition we show how the output from a 3-port network is altered, when conditions of perfect absorption are met but the input waves phase and amplitude vary. In that regard, we propose optimized structures which feature both perfect absorption and perfect transmission at the same frequency by tuning the amplitudes and phases of the input waves.

## Introduction

The absorption of wave energy is a phenomenon which underlies many applications in acoustics and photonics including molecular sensing^1,2^, photodetection^3^, and sound proofing^4,5^. By exploring wave interference, the absorption can become much more efficient or even complete. Indeed, coherent perfect absorbers have been extensively studied the last years in different photonic^6–14^ and acoustic^15–17^ structures. However, the majority of these works deals with two-port systems. Especially in acoustics, the phenomenon of perfect absorption has attracted great attention the last years due to its direct applications to numerous noise reduction problems. Many solutions have been proposed in the low frequency regime based on subwavelength metamaterial designs, by critically coupling^18^ resonant scatterers to the waveguide i.e. by balancing the energy leackage and the internal losses of the resonators. Such studies include the use of membranes^4,16,19–22^, quarter-wavelength structures^23–25^, the concept of slow sound^26–28^ and Helmholtz resonators both in the linear^15,29,30^ and nonlinear^31^ regimes.

Muti-port devices have a great use in applications such as radio frequency (RF) systems and signal/information processing. For example in microwave physics, devices such as power dividers, circulators, filters couplers and multiplexer are commonly used in many RF systems^32,33^. On the other hand, complex photonic circuits have been studied and designed for optical signal processing or computing in integrated optics^34,35^. From theoretical perspective, the study of complex multi-port networks is also a vibrant area of research attracting considerable attention in different fields, including plasmonics^36–39^, quantum transport^40,41^ and acoustics^42–46^.

Although Coherent Perfect Absorption (CPA) has been thoroughly studied both experimentally and theoretically in two-port systems, it is only recently that multi-port devices have been proposed as perfect absorbers. In particular in ref.^47^, in an asymmetric three-port acoustic device it was found that acoustic energy could be channeled from one port to another using a phase mismatch of the input. Additionally adding an additional branch in a standard PT-symmetric electromagnetic waveguide^48,49^, it was shown that it is possible to achieve asymmetric output from this branch when the system is excited from either the loss or the gain side of the main waveguide.

Here, using subwavelength resonators and interferometric control of absorption we illustrate, both theoretically and experimentally, an acoustic perfect absorbing network that operates at different wavelengths, different intensities and relative phases of the input waves. It is found that, in great contrast to the two-port case, there is an infinity of input wave combinations that can be completely absorbed, when the device satisfies CPA conditions. Moreover, we propose optimized 3-port networks which operate both as perfect absorbers and Coherent Perfect Transmitters (CPT), for the same frequency. We show how these systems undergo a transition from CPA to CPT by just tuning the phase and/or the amplitude of the input waves. A high contrast of output to input power ratio is obtained, due to the use of point-like acoustic scatterers. Therefore we believe that utilizing the proposed subwavelength, interferometric control of absorption in multi-port devices and exploring further the role of symmetries could have a strong impact in the field of compact wave devices.

In Section 1, we study the general case of a three port, single-mode, scattering system and obtain the necessary conditions for achieving CPA. The conditions depend both on the device scattering properties and the coherence of the three incoming waves. We show both theoretically and experimentally that an acoustical network composed by three waveguides side-loaded with Helmholtz resonators, can be tuned in order to satisfy these conditions, and CPA is achieved for different type of inputs (asymmetric and symmetric) in the subwavelength frequency regime. In Section 2, we study the interferometric control of the network using different input vectors when the system is in the CPA configuration. We obtain the necessary conditions under which a 3-port network exhibits both CPA and CPT. Using an optimization process, we propose realistic networks satisfying these conditions and verify our results using finite elements 3D simulations.

## Symmetric 3-port system

### Scattering properties

In this work, we study reciprocal and symmetrical 3-port acoustic networks and we focus our analysis on the corresponding scattering matrix which is given by the following equation

1 $$\begin{array}{lllll}(\begin{array}{c}{p}_{1}^{-}\\ {p}_{2}^{-}\\ {p}_{3}^{-}\end{array}) & = & (\begin{array}{lll}r & t & t\\ t & r & t\\ t & t & r\end{array})(\begin{array}{c}{p}_{1}^{+}\\ {p}_{2}^{+}\\ {p}_{3}^{+}\end{array}) & = & S(\begin{array}{c}{p}_{1}^{+}\\ {p}_{2}^{+}\\ {p}_{3}^{+}\end{array})\end{array}.$$

**Figure 1:**
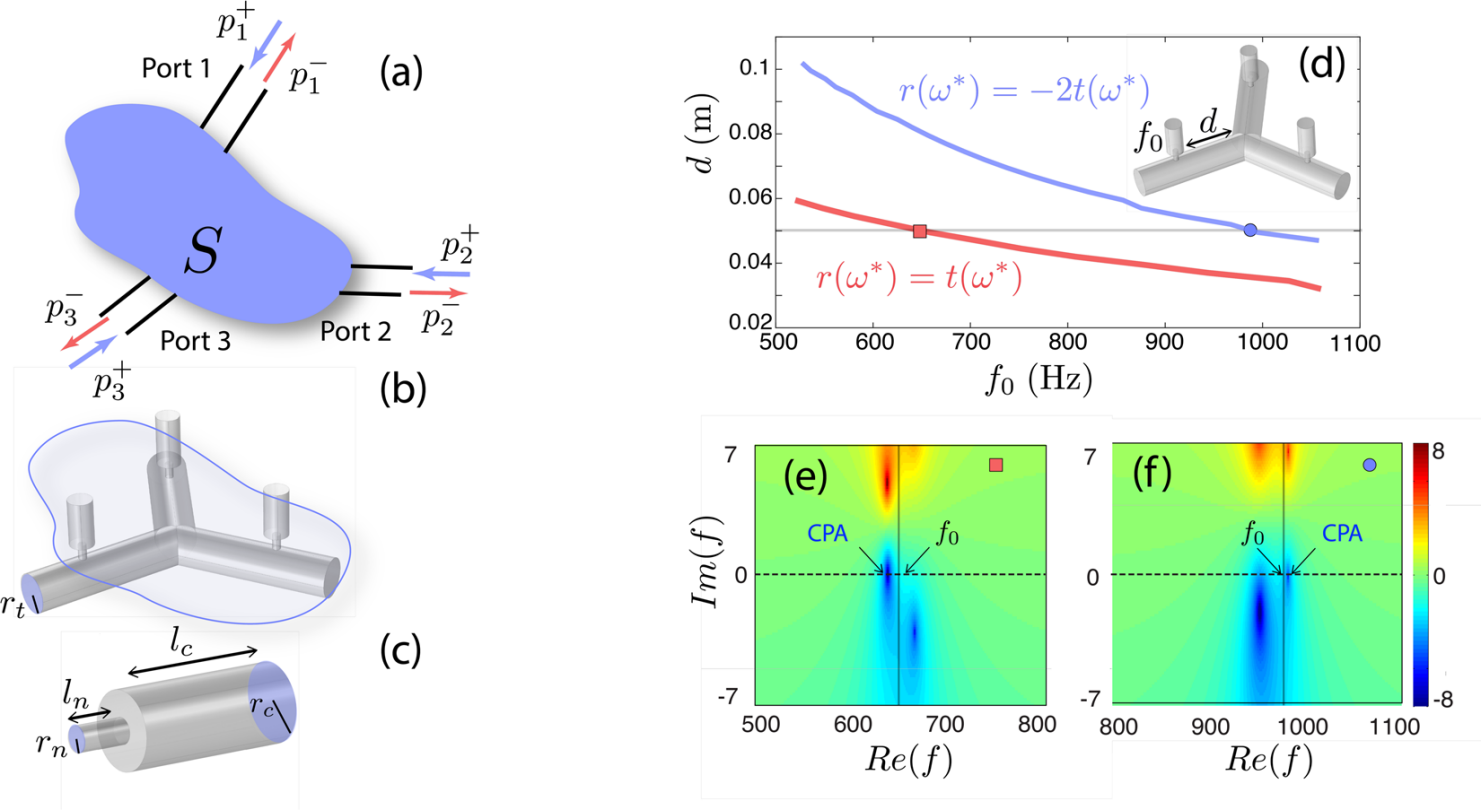
(**a**) A schematic illustration of a general three port system with incoming and outgoing waves at each port. The scattering matrix *S* of the system is assumed to be symmetric, at some frequency range, even if the geometry of the device may be not. (**b**) The symmetric network studied here (not in scale), which is composed by 3 identical cylindrical waveguides with radius *r*_*t*_ = 2.5 × 10^−2^ m, assembled by a Y-shape connection sideloaded with HRs at a distance *d*. (**c**) Details of the HRs composed by a neck with length ${\ell }_{n}=2\times {10}^{-2}$ m, a radius *r*_*n*_ = 0.45 × 10^−2^ m branched to a cylindrical cavity with radius *r*_*c*_ = 1.5 × 10^−2^ m and varying length ${\ell }_{c}$ which is used in order to tune the resonance frequency *f*_0_. (**d**) The upper [lower] curve depicts the symmetric [asymmetric] CPA solutions defined the by Eqs (6 and 7), for varying resonant frequency *f*_0_ and distance *d*. The horizontal line corresponds to the configurations with *d* = 0.05 m. (**e**,**f**) The determinant log(|det(S)|) in the complex frequency plane for the configurations corresponding to the two cases of panel (d) with a (red) square and a (blue) circle respectively.

In Eq. (1) the vectors ${({p}_{1}^{+},{p}_{2}^{+},{p}_{3}^{+})}^{T}\equiv |{\psi }_{{\rm{in}}}\rangle$ and ${({p}_{1}^{-},{p}_{2}^{-},{p}_{3}^{-})}^{T}\equiv |{\psi }_{{\rm{out}}}\rangle$ describe the incoming and outgoing waves respectively as shown in Fig. 1(a). The frequency dependent coefficients *r* and *t*, correspond to the reflection and transmission when only one port is excited as explained in details in the Appendix. Note that the S-matrix as given by Eq. (1) is a *symmetric circulant* matrix which stemms both from the geometrical symmetry of the 3-port network and from the fact that we consider a reciprocal system.

Furthermore the eigenvalue problem associated with the S-matrix is given by

2 $${\rm{\det }}(S-\lambda I)=\mathrm{0,}$$

where the eigenvalues are found to be:

3 $${\lambda }_{0}=r+2t,\,{\rm{and}}\,{\lambda }_{1}={\lambda }_{2}=r-t,$$

and the corresponding orthonormal eigenvectors are given by:

4 $$|{u}_{0}\rangle =\frac{1}{\sqrt{3}}{\mathrm{(1},1,\mathrm{1)}}^{T},\,|{u}_{1}\rangle =\frac{1}{\sqrt{3}}{(\mathrm{1,}{e}^{2i\pi /3},{e}^{-2i\pi \mathrm{/3}})}^{T},\,|{u}_{2}\rangle =\frac{1}{\sqrt{3}}{(1,{e}^{-2i\pi \mathrm{/3}},{e}^{2i\pi \mathrm{/3}})}^{T}\mathrm{.}$$

It is interesting to note here that, the eigenvectors of a circulant matrix are always the same and are independent of both the physical system (particular form of *r* and *t*) and the frequency.

The above eigenanalysis is useful for the study of CPA since if an eigenvalue *λ*_*i*_ of *S* is found to be zero, then using the corresponding eigenvector $|{u}_{i}\rangle$ as input in Eq. (1) will result in zero output, thus perfect absorption. To quantify the absorption efficiency of the 3-port network below we use the ratio of total output to input power defined as

5 $${\rm{\Theta }}=\frac{\sum _{i}{|{p}_{i}^{-}|}^{2}}{\sum _{i}{|{p}_{i}^{+}|}^{2}}=\frac{\sum _{i}{\lambda }_{i-1}^{2}{|{c}_{i-1}|}^{2}}{\sum _{i}{|{c}_{i-1}|}^{2}}\,i=\mathrm{1,}\,\mathrm{2,}\,3.$$

In the last expression we have used the fact that, since the eigenvectors $|{u}_{i}\rangle$ form a complete basis, we may write any input vector as a sum of $|{u}_{i}\rangle$, i.e. $|{\psi }_{{\rm{in}}}\rangle ={\sum }_{i}{c}_{i}|{u}_{i}\rangle$.

### Coherent Perfect Absorption

According to the eigenanalysis, a 3-port network exhibits CPA if any of the eigenvalues given by Eq. (3) becomes zero at some particular frequency f*. We first consider the case of *λ*_0_ = 0, which leads to the following condition for the scattering coefficients

6 $$r=-\,2t\equiv {r}_{s}\mathrm{.}$$

If Eq. (6) is satisfied, then a symmetric input of the form $|{u}_{0}\rangle$ [Eq. (4)] will be completely absorbed. Below we refer to the combination of Eq. (6) and an input $|{u}_{0}\rangle$ as *symmetric* CPA.

On the other hand, by setting the degenerate eigenvalues *λ*_1,2_ = 0 we obtain a different CPA condition

7 $$r=t\equiv {r}_{a}\mathrm{.}$$

When Eq. (7) is satisfied, an input wave in the form of the asymmetric vectors $|{u}_{1}\rangle$ or $|{u}_{2}\rangle$ is completely absorbed. Moreover, since the system is linear, any input in the form of $|{\psi }_{{\rm{in}}}\rangle =\alpha |{u}_{1}\rangle +\beta |{u}_{2}\rangle$ will also be completely absorbed and since *α* and *β* are free parameters this leads to infinite combinations of asymmetric input waves. This is in great contrast with two port systems where CPA is achieved either by in-phase or out-of phase incoming waves. Here, the additional port acts as a “control” port which, whenever Eq. (7) is satisfied, is able to lead the 3-port network to CPA. We further on refer to this combination of condition (7) and the inputs as *asymmetric* CPAs.

The conditions to achieve CPA given by Eqs (6) and (7) require that the transmission and reflection coefficients are specifically tuned. A popular way to achieve this is to use Fano resonance phenomena by employing resonant scatterers, especially regarding subwavelength manipulation of waves. In acoustics the most prominent example of such a scatterer is the Helmholtz resonator (HR), which has been intensively studied in the context of wave absorption and acoustic metamaterials. Due to the presence of HRs sided loaded to the waveguide, the strong interference around the resonance frequency greatly modifies both the transmission and the reflection coefficients and allows us to satisfy the CPA conditions.

The particular system under consideration in this work is schematically shown in Fig. 1(b,c). We consider all HRs to have identical resonance frequencies *f*_0_ and to be placed at the same distance *d* from the center of the device. In the low frequency regime, assuming only the propagation of the plane mode of the waveguides and approximating the HRs as point scatterers we can calculate *t* and *r* using the standard transfer matrix method. The analytic calculations of *t* and *r* are detailed in the section Method.

In order to obtain the configurations which exhibit CPA, we calculate *r* and *t* by scanning the parametric space (*f*_0_, *d*) and monitor when the conditions of Eqs (6) and (7) are satisfied. The rest of the parameters are fixed to the experimental values given in the caption of Fig. 1. The results are shown in Fig. 1(d) where the red (lower) curve corresponds to the asymmetric and the blue (upper) line depicts the symmetric CPA. Another way to illustrate the occurrence of the CPA is through the complex frequency plane of the determinant of the scattering matrix *S*. In this representation, by scanning the parametric space (*f*_0_, *d*) the zeros of the determinant are moving while exactly at the CPA condition, one of those crosses the real axis. Note here, that another way to achieve CPA would be to choose a particular point of the parametric space (*f*_0_, *d*) (namely fixing the geometry of the system) and increase the viscothermal losses of the system.

In Fig. 1(e,f), we plot the log |det *S*| for two different configurations which satisfy Eqs (6) and (7) respectively. It is directly shown that in both cases, one zero of the determinant is located on the real axis. As indicated in Fig. 1(e,f), the operating frequency for the CPA devices is near (but not the same) to the resonant frequency of the HRs due to the interaction of the resonances of each HR through the waveguides.

### Experimental observation of acoustic CPA

**Figure 2:**
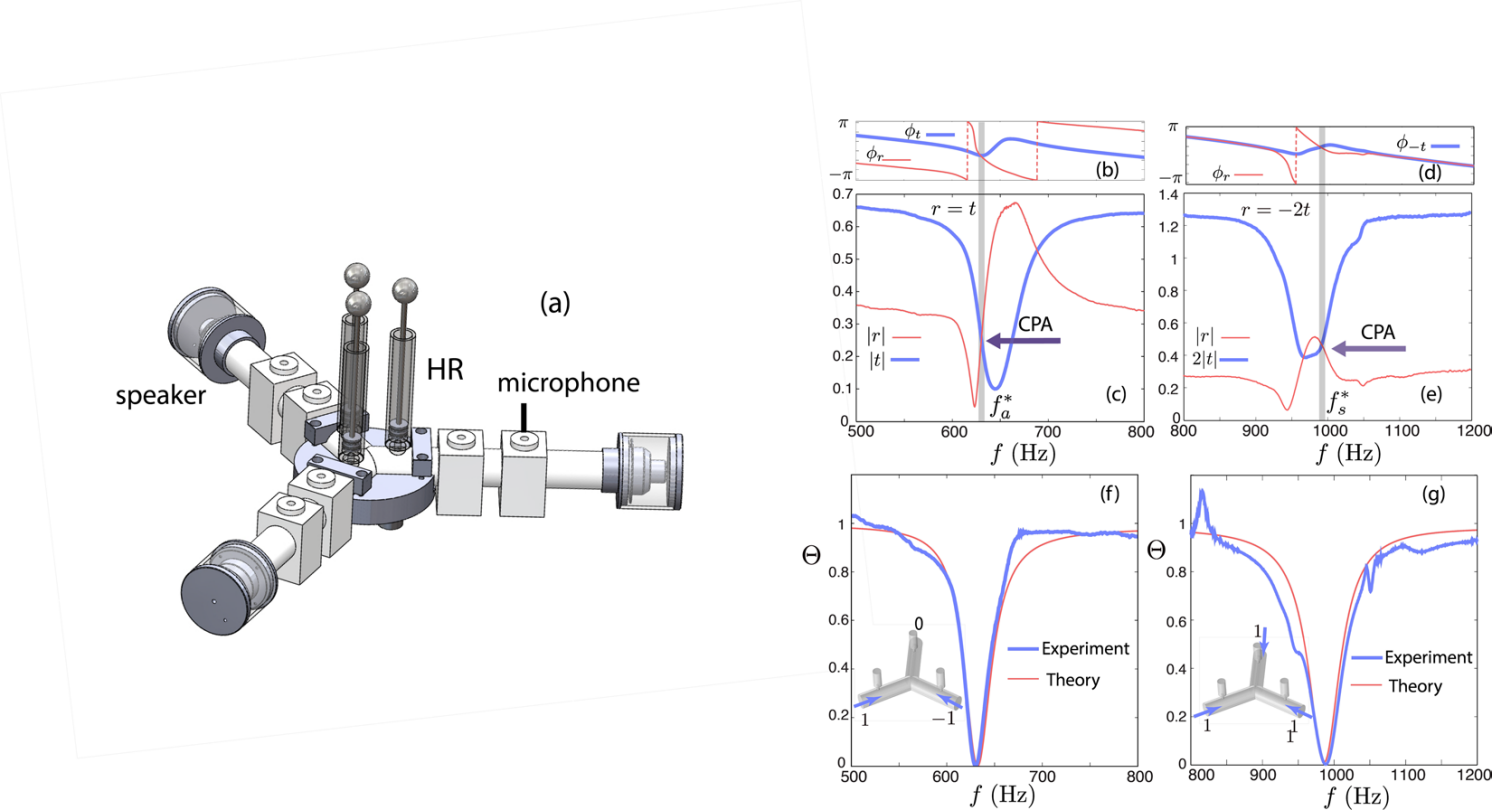
(**a**) View of the experimental device. (**b**,**c**) The phase and absolute value of the experimentally measured reflection and transmission coefficients *t* and *r*, as a function of frequency, for the configuration which exhibits an asymmetric CPA at ${f}_{a}^{\ast }=630$ Hz. The resonance frequency of the HRs is *f*_0_ = 645 Hz corresponding to a cavity length *l*_*c*_ = 0.02 m. CPA is ensured since both the real and the imaginary part of *r* and *t* are equal for this frequency (vertical gray line). (**d**,**e**) The phase and absolute value of the experimentally measured reflection and transmission coefficients *r* and −2*t*, as a function of frequency, for the configuration which exhibits a symmetric CPA at ${f}_{s}^{\ast }=988$ Hz. The resonance frequency of the HRs is *f*_0_ = 975 Hz corresponding to a cavity length *l*_*c*_ = 0.89 × 10^−2^ m. CPA appears when the two curves of both the real and imaginary part become equal, indicated by the vertical gray line. (**f**,**g**) The output to input power ratio Θ as a function of frequency for the asymmetric and the symmetric CPA respectively. The thick (thin) line corresponds to the experimental (theoretical) measurement. The insets depict the eigenvector used in order to obtain the curves in each case.

In this Section, we experimentally verify the analytical results of Fig. 1(d) in an acoustic network with *d* = 0.05 m. We use two sets of HRs with different resonant frequencies *f*_0_ corresponding to the asymmetric and symmetric CPA as indicated respectively by the square and the circle in Fig. 1(d). In our experiments, the 3-ports network is driven with plane waves produced by loudspeakers (AURA NS3-193-8A 3 inch) placed at the end of each port (see Fig. 2(a)). The system is considered to be symmetric as long as the driving frequencies are below the first cut-of frequency of the waveguides such that no higher modes are propagating. To determine experimentally the reflection and transmission coefficients, a pair of microphones (1/2 inch B&K) connected to each waveguide is employed, allowing the measurement of both the forward ${p}_{i}^{+}$ and backward ${p}_{i}^{-}$ waves in each waveguide^50,51^. Using the definition of the scattering matrix Eq. (1) and the measured values of the incoming and outgoing waves, we can directly obtain *t* and *r*.

We perform measurements for a range of driving frequencies between 400 Hz to 1200 Hz using a sweep sine function, and the results are shown in Fig. 2. Panels (b and c) correspond to the asymmetric CPA. The particular shape of both transmission and reflection coefficients is due to strong interference originating from the large reflection of each resonator around the resonance frequency.

Consequently, for this setting at frequency ${f}_{a}^{\ast }=630$ Hz the required condition for the asymmetric CPA *r* = *t* is fulfilled as indicated by the vertical gray line. Similarly, for the configuration corresponding to the symmetric case, as shown in Fig. 2(d,e) the condition *r* = −2*t*, is also fulfilled at the CPA frequency ${f}_{s}^{\ast }=988$ Hz (vertical gray line). The frequency of the CPA is not equal to the resonance frequency of the HRs i.e. *f*_0_ = 645 Hz and *f*_0_ = 975 Hz for the asymmetric and symmetric case respectively, as explained in the previous section.

By measuring the complex coefficients *r* and *t* we have experimentally determined the scattering matrix of the 3-port network for the two different configurations for the frequency range of interest. We can thus now quantify the ability of our setup to completely absorb an input wave at the CPA frequency, by considering an asymmetric input vector ${({p}_{1}^{+},{p}_{2}^{+},{p}_{3}^{+})}^{T}={\mathrm{(1,}-1,0)}^{T}$ [inset of Fig. 2(f)] and the experimentally obtained scattering matrix in Eq. (1). Then from the corresponding output we may calculate Θ by its definition from Eq. (5). The result is shown in Fig. 2(f) with the thick solid line, where at the CPA frequency we obtain an almost perfect absorption with Θ ≈ 5 × 10^−4^. The experimental result is in a good agreement with the theoretical prediction [thin solid line in Fig. 2(f)] calculated using the transfer matrix method.

We perform the same analysis for the network with the symmetric CPA. In particular, considering an input of the form ${({p}_{1}^{+},{p}_{2}^{+},{p}_{3}^{+})}^{T}={(1,1,1)}^{T}$ [inset of Fig. 2(g)] and using the experimental values of *r* and *t* [see Fig. 2(d,e)] we obtain Θ for a frequency range between 800 Hz to 1200 Hz. The result is shown with the thick solid line in Fig. 2(g), and in this case the system reaches a value of Θ ≈ 10^−3^ at ${f}_{s}^{\ast }$. The peak at Fig. 2(g) appearing around 850 Hz is due to the limitation of the two microphones method. The latter fails in the vicinity of the resonance of the cavity created by the main waveguide closed at one end by the loudspeaker. The second peak around 1 kHz is due to small structural imperfections of our device. The comparison with the theoretical result illustrated with the thin line Fig. 2(g) shows a good agreement between the two, close to the CPA frequency. At this frequency, the wavelength is 0.53 m and 0.34 m for asymmetric and symmetric CPA respectively. This is much larger than the diameter of the neck of the HRs (2*r*_*n*_ = 0.9 × 10^−2^ m) and it is for this reason that our analytical model based on point scatterer approximation for the HRs is in agreement with the experimental observations. Finally, to characterize the subwavelength efficiency of our system we use the ratio *λ*/2*d*. For the case of asymmetric CPA, *λ*/2*d* = 5.4 while for the symmetric CPA, *λ*/2*d* = 3.4.

## Interferometric control of the 3-port network

Up to this point we have shown that when a 3-port network is tuned to satisfy either Eqs (6) or (7) then it completely absorbs certain combinations of input waves from each port, corresponding to the *S* matrix eigenvectors (or their linear combinations). Here we study the behavior of such a tuned network, varying the input waves such that we deviate from the perfectly absorbed eigenvectors and the device transmits some amount of energy. In fact our goal is to identify the conditions under which a 3-port network acts both as perfect absorber (Θ = 0) and a perfect transmitter (Θ = 1) at the same frequency.

According to Eq. (5), an input vector in the form of an eigenvector of *S*, i.e. $|{\psi }_{{\rm{in}}}\rangle =|{u}_{i}\rangle$ will result in ${\rm{\Theta }}={\lambda }_{i}^{2}$ as output to input ratio. If for the same parameter values and at the same frequency there is an eigenvalue *λ*_*i*_ = 0 and one with *λ*_*j*≠*i*_ = 1 then the device exhibits both CPA and CPT. This constrain defines a pre-described value for the coefficients *r* and *t* which is different depending whether Eqs (6) or (7) is satisfied.

### From asymmetric CPA to symmetric CPT

We first consider the case when Eq. (6) is satisfied and thus *λ*_1,2_ = 0 leading to CPA with an asymmetric input $|{\psi }_{{\rm{in}}}\rangle =\alpha |{u}_{1}\rangle +\beta |{u}_{2}\rangle$. Demanding also *λ*_0_ = 1 leads to *r*_*a*_ = *t* = 1/3 [see Eq. (3)] and for the same 3-port network a symmetric input $|{\psi }_{{\rm{in}}}\rangle =|{u}_{0}\rangle$ results in CPT. Let’s note that, for our experimental device, as illustrated in Fig. 2(b), the measured value of the reflection coefficient is $|{r}_{a}^{{\rm{e}}xp}|=0.25$ and thus CPT cannot be reached.

In order to check if a realistic device that satisfying both the conditions for CPA and CPT can be found, we use an optimization algorithm. We first choose a prescribed operating frequency close to asymmetric CPA experimental value ${f}_{a}^{\ast }=630$ Hz and then we optimize the geometrical characteristics of the device to achieve the prescribed condition *r*_*a*_ = *t* = 1/3 at ${f}_{a}^{\ast }$ (*r* and *t* are calculated using the analytical expressions from the transfer matrix method). The optimization method converges up to 99% with ${r}_{a}^{{\rm{op}}}=0.33$ and the resulting geometrical parameters of the optimized device are given in the Method section.

To study the transition from CPA to CPT for the optimized network, we calculate Θ using an input vector of the form

8 $${u}_{{I}_{a}}={(1,-{e}^{i\varphi },\gamma )}^{T}\mathrm{.}$$

Here, *γ* is a real number characterizing the ratio between the amplitudes of the incoming waves and *ϕ* denotes the phase difference from the CPA eigenvector (defined by *γ* = 0 and *ϕ* = 0). Using Eqs (1), (7) and (5) we find that

9 $${\rm{\Theta }}(\gamma ,\,\varphi )=3{|{r}_{a}|}^{2}(1+\frac{\mathrm{2(}\gamma -\,\cos \,\varphi \mathrm{(1}+\gamma ))}{2+{\gamma }^{2}})\mathrm{.}$$

**Figure 3:**
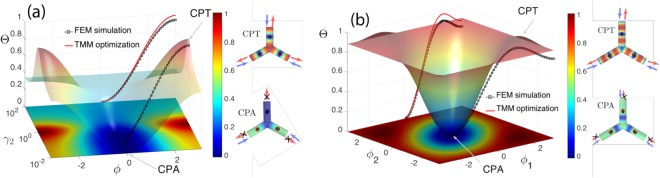
(**a**) The output to input power ratio Θ as a function of *γ* and *ϕ* using the input vector ${u}_{{I}_{a}}$, when the system is in the optimal configuration for asymmetric CPA with ${r}_{a}^{{\rm{op}}}=0.33$, as given by Eq. (9). At the (*ϕ*, Θ) plane we show the result obtained with the FEM simulation (squares) and with the result of Eq. (9) (solid line), along the trajectory *γ* = *ϕ*/*π* with *γ* ∈ [0, 1]. On the righthand side we plot the field distribution of the absolute pressure in the network at *γ* = *ϕ* = 0 (CPA) and at *γ* = *ϕ*/*π* = 1 (CPT) as obtained by the FEM simulation. (**b**) The output to input power ratio Θ as a function of *ϕ*_1_ and *ϕ*_2_ using the input vector ${u}_{{I}_{s}}$ with *γ*_1,2_ = 1, when the system is in the optimal configuration for symmetric CPA with at ${r}_{s}^{{\rm{op}}}=0.657$, as given by Eq. (9). In the (*ϕ*_1_, Θ) plane we show the result obtained with the FEM simulation (squares) and with the result of Eq. (9) (solid line), along the trajectory *ϕ*_1_ = −*ϕ*_2_ with *ϕ*_1_ ∈ [0, *π*]. On the righthand side we plot the field distribution of the absolute pressure in the network at *ϕ*_1,2_ = 0 (CPA) and at *ϕ*_1_ = −*ϕ*_2_ = 2*π*/3 (CPT) as obtained by the FEM simulation.

In Fig. 3(a) we plot the parameter Θ as a function of *γ* and *ϕ*. We identify the CPA point at *γ* = *ϕ* = 0 and the CPT points at *γ* = ±*ϕ*/*π* = 1. From Eq. (9) and as illustrated in Fig. 3(a) the CPA and CPT points, are the extrema of this function. As such, small deviations around these points will lead to a *quadratic sensitivity* of the two phenomena. The solid line in the (*ϕ*, Θ) plane shows Θ for a particular trajectory defined by *γ* = *ϕ*/*π* where *γ* ∈ [0, 1], which starts at CPA (*γ* = 0) and ends at CPT (*γ* = 1).

In addition, to verify the efficiency of the optimized device in the full 3D space, we use a 3D FEM simulations and study the corresponding geometry for the trajectory *γ* = *ϕ*/*π* with ${u}_{{I}_{a}}$. The result of the FEM simulation is shown with the black square lines both on top of the 3D contour and on the (*ϕ*, Θ) plane. There is a good agreement between the two results, verifying our model using the transfer matrix method. The 3D network is able to achieve an almost total absorption with an output ratio Θ ≈ 10^−3^, and an almost perfect transmission with Θ = 0.93.

### From symmetric CPA to asymmetric CPT

For the case where Eq. (7) is satisfied and thus *λ*_0_ = 0, the network exhibits CPA for symmetric inputs of the form $|{\psi }_{{\rm{in}}}\rangle =|{u}_{0}\rangle$. Here the additional condition for CPT is *λ*_1,2_ = 1 which leads to *r*_*s*_ = −2*t* = 2/3. Then the device will also completely transmit asymmetric inputs of the form $|{\psi }_{{\rm{in}}}\rangle =\alpha |{u}_{1}\rangle +\beta |{u}_{2}\rangle$. For this case our experimental setup featuring a value $|{r}_{s}^{\exp }|=0.52$ is not able to reach CPT.

Thus, an optimization scheme is again used to obtain a device that achieves both CPA and asymmetric CPT. We choose to optimize at a frequency close to the experimental symmetric CPA frequency ${f}_{s}^{\ast }=988$ Hz. The optimization method converges up to 98.5% corresponding to ${r}_{a}^{{\rm{op}}}=0.657$ and the parameters of the optimized device are given in the Method section.

In a similar way as in the asymmetric case, we study the dependence of Θ as a function of two parameters, using the input vector

10 $${u}_{{I}_{s}}={(\mathrm{1,}{e}^{i{\varphi }_{1}},{e}^{i{\varphi }_{2}})}^{T},$$

where *ϕ*_1,2_ quantify the phase differences from the CPA eigenvector. Using Eqs (1) and (5) we obtain that

11 $${\rm{\Theta }}({\varphi }_{\mathrm{1,2}})=\frac{3}{2}{|{r}_{s}|}^{2}(1-\frac{\cos \,{\varphi }_{1}+\,\cos \,{\varphi }_{2}+\,\cos ({\varphi }_{1}-{\varphi }_{2})}{3})\mathrm{.}$$

The dependence of Θ on the two phase differences is plotted in Fig. 3(b). We observe that the maximum of Θ as we discern from the CPA eigenvector, is obtained when the two phase differences satisfy *ϕ*_1_ = −*ϕ*_2_ = 2*π*/3, i.e. when $|{u}_{{I}_{S}}\rangle =|{u}_{1,2}\rangle$. In this case also, as illustrated in Fig. 3(b), the CPA and CPT points are the extrema of this function and small deviations around these points imply a *quadratic sensitivity* of the two phenomena.

The optimized device’s scattering properties are also verified by means of 3D FEM simulations using an input vector of the form of Eq. (10) and following the trajectory *ϕ*_1_ = −*ϕ*_2_ with *ϕ*_1_ ∈ [0, *π*]. The result of the FEM simulations are shown on the (*ϕ*_1_, Θ) plane and on the 3D plot by the black square lines and are found to be in a good agreement with the 1D analytical model. The device is able to reach CPA with an input output ratio Θ = 10^−4^ and a strong transmission with Θ = 0.91. Let’s note that in this case the high output contrast of the network can be controlled only by the relative phase of the inputs and not the amplitudes.

## Discussion

We conclude by discussing the ability of a 3-port network to exhibit a very large output contrast (from CPA to CPT) at the same frequency. The physical mechanism of CPA is based on the balance of the losses stemming from the resonator and the leakage rate of energy from the resonator to the waveguide. This is achieved by operating close to the resonance frequency where the losses from the HR are strong. Thus, in order to achieve CPT we need to annihilate the effect of losses from the HRs, and this is possible by engineering the input waves and create a destructive interference pattern at the positions of the HRs, which in the low frequency regime act almost like point scatterers. In fact, the disagreement between the analytical 1D model and the FEM simulations in the projections in Fig. 3(a,b) close to the CPT point, stems exactly from the fact that in the analytical model HRs are considered as points, while in the FEM they are small but finite. However the mechanism for CPT is the same, as illustrated in the right panels of Fig. 3(a,b) for the CPT pressure profiles which acquire a minimum at the position of the resonator.

In Section II we have found the conditions to achieve coherent perfect absorption in a symmetric 3-port network, using the eigenvalues and eigenvectors of the corresponding *S* matrix. The conditions are directly related to the values of the transmission (*t*) and the reflection (*r*) coefficients. It is found that the condition *r* = *t* leads to an infinity of asymmetric inputs which are perfectly absorbed (asymmetric CPA) while for *r* = −2*t* a symmetric input of in-phase and equal amplitude waves from each port is perfectly absorbed (symmetric CPA). This is in great contrast with the two port case where (for a particular device) only one input is able to achieve CPA. The above conditions, are experimentally observed in an acoustic 3-port network composed of three connected waveguides sideloaded by Helmholtz resonators in the low frequency audible range. In Section III we have studied how the output of the system is affected when the input waves deviate from the corresponding CPA inputs. In particular we have shown that a 3-port network can exhibit both perfect absorption and perfect transmission at the same frequency if an additional constrain is imposed. The latter dictates a value of *r* = *t* = 1/3 for the case of an asymmetric CPA (leading to a symmetric CPT), and *r* = −2*t* = 2/3 for a symmetric CPA (thus an asymmetric CPT). The dependence of the output to input power ratio on the relative amplitude and phases of the input vectors is found analytically. Using this expression we find that deviations from CPA and CPT are extrema of this function and thus are quadratically depended on small amplitude and phase mismatches. Using an optimization algorithm and the analytical expressions of the *S* matrix elements, we further provide particular examples of acoustic networks able to achieve both CPA and CPT (both symmetric and asymmetric). The optimization method never converged 100 % since both our model (and the realistic system) exhibits distributed losses and for any finite length of propagation some energy is always lost. Finally, using FEM 3D simulation of the optimized configurations we have confirmed the transition from perfect absorption to a nearly perfect transmission.

The additional control over perfect absorption and the ability to go from CPA to CPT using point scatterers, indicate that the 3-port system can be used as a unit cell to construct complex networks with prescribed wave scattering properties. Our theoretical results can be directly generalized for symmetric N-port systems, and using them build periodic networks with different characteristics. Additionally, 3-port networks which are asymmetric, exhibit more degrees of freedom and could provide the means for extra control, such as directional propagation from selected ports.

## Method

### Determination of the elements of the scattering matrix

**Figure 4:**
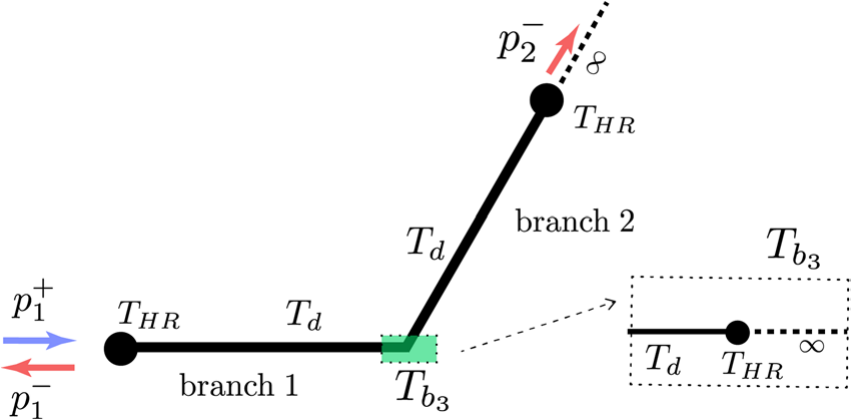
Schematic of the 3-port system for the determination of the reflection and transmission coefficients by the Transfert Matrix Method. *T*_*HR*_ describes the scattering by the side-loaded HR. *T*_*d*_ illustrates the propagation along the waveguide of length *d*. ${T}_{{b}_{3}}$ describes the influence of the third branch composed by a infinite waveguide side-loaded by a HR at the distance *d* from the connection.

We consider the following 2-port scattering problem

12 $$(\begin{array}{c}{p}_{1}^{-}\\ {p}_{2}^{-}\end{array})=(\begin{array}{cc}r & t\\ t & r\end{array})(\begin{array}{c}{p}_{1}^{+}\\ {p}_{2}^{+}\end{array})={S}_{2\times 2}(\begin{array}{c}{p}_{1}^{+}\\ {p}_{2}^{+}\end{array})$$

which described the reflection and transmission from port 1 to port 2 *in the presence of* port 3, as shown in Fig. 4. These coefficients can then be used to describe the full 3 by 3 scattering problem as defined in Eq. (1), due to the symmetry of the network. To compute *r* and *t* we use the Transfer Matrix Method (TMM) which utilizes the continuity of the pressure field and the conservation of mass. The corresponding matrix equation is given by

13 $$(\begin{array}{c}{P}_{1}\\ {U}_{1}\end{array})=T(\begin{array}{c}{P}_{2}\\ {U}_{2}\end{array})=(\begin{array}{cc}{T}_{11} & {T}_{12}\\ {T}_{21} & {T}_{22}\end{array})(\begin{array}{c}{P}_{2}\\ {U}_{2}\end{array})\mathrm{.}$$

Here, ${P}_{i}={p}_{i}^{+}+{p}_{i}^{-}$ is the total pressure and ${U}_{i}=S{\partial }_{{n}_{i}}{P}_{i}/(i\omega \rho )$ is the acoustic flux velocity parallel to the vector *n*_*i*_, normal to the waveguide section at the *i*-th port. We consider sound propagation in air with density *ρ* and *ω* denotes the angular frequency.

Eq. (13) is obtained by considering an incident wave from port 1 and assuming no backward waves for the two ports remaining (no input and anechoic end) as illustrated in the Fig. 4. Then by rearranging Eq. (13) we obtain the matrix elements of Eq. (12) as follows.

14 $$r=\frac{{T}_{11}+{T}_{12}/{Z}_{c}-{T}_{21}{Z}_{c}-{T}_{22}}{{T}_{11}+{T}_{12}/{Z}_{c}+{T}_{21}{Z}_{c}+{T}_{22}},\,t=\frac{2}{{T}_{11}+{T}_{12}/{Z}_{c}+{T}_{21}{Z}_{c}+{T}_{22}}$$

where *Z*_*c*_ = *ρc*/*S* is the characteristic impedance of the waveguide of cross-section *S*. The elements of the matrix $T={T}_{HR}{T}_{d}{T}_{{b}_{3}}{T}_{d}{T}_{HR}$ are explained in the following. The transfer matrix *T*_*HR*_, corresponds to the HR loading the waveguide and is given by

15 $${T}_{HR}=(\begin{array}{cc}1 & 0\\ {Y}_{HR} & 1\end{array})$$

where *Y*_*HR*_ is the entrance admittance of the HR considered as a point scatterer. The expression for the admittance can be found for example in ref.^15^, where detailed information about the viscothermal losses are also included. *T*_*d*_ is the transfer matrix for the propagation along the waveguide of length *d* and is given by

16 $${T}_{d}=(\begin{array}{cc}\cos (kd) & i{Z}_{c}\sin (kd)\\ i/{Z}_{c}\sin (kd) & \cos (kd)\end{array})$$

where *k* is the wavenumber. ${T}_{{b}_{3}}$ describes the influence of the third branch of the network (see Fig. 4), and takes the following form

17 $${T}_{{b}_{3}}=(\begin{array}{cc}1 & 0\\ {Y}_{{b}_{3}} & 1\end{array})$$

where ${Y}_{{b}_{3}}$ is the entrance admittance of the branch 3 given by

18 $${Y}_{{b}_{3}}=\frac{1}{{Z}_{c}}\frac{\cos (kd\mathrm{)(1}+{Z}_{c}{Y}_{HR})+i\,\sin (kd)}{\cos (kd)+i{Z}_{c}\,\sin (kd\mathrm{)(1}+{Y}_{HR})}\mathrm{.}$$

In our calculations we consider the effect of losses in the waveguide and in the HRs using the Zwikker and Kosten model^52^ which includes an imaginary part in the wavenumber and in the characteristic impedances of the waveguides. Namely, we replace the wave number and the impedances by the expressions

19 $$k=\frac{2\pi f}{c}(1+\frac{\beta }{s}(1+(\gamma -1)/\xi ))\,\mathrm{and}\,Z=\frac{\rho c}{S}(1+\frac{\beta }{s}(1-(\gamma -1)/\xi ))$$

by setting *s* = *R*/*δ* where *R* is the radius of the considered waveguide, and $\delta =\sqrt{2\mu \mathrm{/(2}\pi \rho \,f)}$ is the viscous boundary layer thickness, with *μ* being the viscosity of air. *c* is the celerity of the wave, *ρ* is the mass density of air and $\xi =\sqrt{{P}_{r}}$ with *P*_*r*_ the Prandl number at atmospheric pressure; $\beta =(1-i)/\sqrt{2}$ and *γ* = 1.4, the heat capacity ratio of air.

### Optimization

From Eq. 14, we obtain the reflection and transmission coefficients as a function of all the system’s geometrical parameters *i.e*. ${r}_{t},\,{\ell }_{n},\,{r}_{n},\,{r}_{c},\,{\ell }_{c},\,d,$ (see Fig. 1) and of the operating frequency *f*. In order to find the device achieving both CPA and CPT, the reflection and transmission coefficients should satisfy Eqs (6 and 7) with the additional constraints *r* = 2/3 [*r* = 1/3]. To obtain solutions satisfying this condition, a numerical optimization based on simplex derivative free method Nelder-Mead^53^ with 6 optimization parameters (fixing the operating frequency) under experimentally and physically reasonable constraints is used.

### Numerical computation

The 3D numerical simulations are conducted with the commercial finite element method (FEM) @Comsol. In the FEM model, the effective expressions for the complex wavenumber and impedances of each part of the system are used to take into account the viscothermal losses. For the asymmetric case, the geometrical parameters of the system are *l*_*c*_ = 0.289 m, *r*_*c*_ = 0.022 m, *l*_*n*_ = 0.049 m, *r*_*n*_ = 0.008 m, *r*_*t*_ = 0.045 m and *d* = 0.136 m. For the symmetric case the parameters are *l*_*c*_ = 0.36 m, *r*_*c*_ = 0.02 m, *l*_*n*_ = 0.036 m, *r*_*n*_ = 0.01 m, *r*_*t*_ = 0.046 m and *d* = 0.17 m.
